# Common *ALDH2* genetic variants predict development of hypertension in the SAPPHIRe prospective cohort: Gene-environmental interaction with alcohol consumption

**DOI:** 10.1186/1471-2261-12-58

**Published:** 2012-07-29

**Authors:** Yi-Cheng Chang, Yen-Feng Chiu, I-Te Lee, Low-Tone Ho, Yi-Jen Hung, Chao A Hsiung, Thomas Quertermous, Timothy Donlon, Wei-Jei Lee, Po-Chu Lee, Che-Hong Chen, Daria Mochly-Rosen, Lee-Ming Chuang

**Affiliations:** 1 Department of Internal Medicine, National Taiwan University Hospital, Taipei, Taiwan; 2 Genomics Research Center, Academia Sinica, Taipei, Taiwan; 3 Division of Biostatistics and Bioinformatics, Institute of Population Health Sciences, National Health Research Institutes, Zhunan, Taiwan; 4 Division of Endocrinology and Metabolism, Department of Internal Medicine, Taichung Veterans General Hospital, Taichung, Taiwan; 5 Department of Medical Research and Education, Taipei Veterans General Hospital, Taipei, Taiwan; 6 Section of Endocrinology and Metabolism, Department of Medicine, Taipei Veterans General Hospital, Taipei, Taiwan; 7 Faculty of Medicine, School of Medicine, National Yang-Ming University, Taipei, Taiwan; 8 Division of Endocrinology & Metabolism, Tri-Service General Hospital, National Defense Medical Center, Taipei, Taiwan; 9 Division of Cardiovascular Medicine, Falk Cardiovascular Research Building, Stanford University School of Medicine, Stanford, CA, USA; 10 Kuakini Medical Center, Honolulu, HI, USA; 11 Department of Surgery, Min-Sheng Hospital, Taoyuan, Taiwan; 12 Department of General Surgery, National Taiwan University Hospital, Taipei, Taiwan; 13 Department of Chemical and Systems Biology, School of Medicine Stanford University, Stanford, CA, USA; 14 Graduate Institute of Clinical Medicine, National Taiwan University College of Medicine, Taipei, Taiwan

**Keywords:** *ALDH2*, Hypertension, SNP, Chinese

## Abstract

**Background:**

Genetic variants near/within the *ALDH2* gene encoding the mitochondrial aldehyde dehydrogenase 2 have been associated with blood pressure and hypertension in several case–control association studies in East Asian populations.

**Methods:**

Three common tag single nucleotide polymorphisms (tagSNP) in the *ALDH2* gene were genotyped in 1,134 subjects of Chinese origin from the Stanford Asia-Pacific Program for Hypertension and Insulin Resistance (SAPPHIRe) family cohort. We examined whether the *ALDH2* SNP genotypes predicted the development of hypertension in the prospective SAPPHIRe cohort.

**Results:**

Over an average follow-up period of 5.7 years, carriers homozygous for the rs2238152 T allele in the *ALDH2* gene were more likely to progress to hypertension than were non-carriers (hazard ratio [HR], 2.88, 95% confidence interval [CI], 1.06-7.84, *P* = 0.03), corresponding to a population attributable risk of ~7.1%. The risk associated with the rs2238152 T allele were strongest in heavy/moderate alcohol drinkers and was reduced in non-drinkers, indicating an interaction between *ALDH2* genetic variants and alcohol intake on the risk of hypertension (*P* for interaction = 0.04). The risk allele was associated with significantly lower *ALDH2* gene expression levels in human adipose tissue.

**Conclusion:**

*ALDH2* genetic variants were associated with progression to hypertension in a prospective Chinese cohort. The association was modified by alcohol consumption.

## Background

The *ALDH2* gene encodes the mitochondrial aldehyde dehydrogenase 2, a critical enzyme involved in alcohol metabolism. After alcohol ingestion, ethanol is first oxidized to acetaldehyde by alcohol dehydrogenase and subsequently converted to acetic acid by ALDH2 [1]. A substantial proportion of the East Asian population carries the rs671 A allele of the *ALDH2* gene causing the E487K mutation (glutamate at codon 487 replaced by lysine) [2,3]. A marked elevation of circulating acetaldehyde after alcohol ingestion is observed in carriers with mutant alleles [4]. Acetaldehyde is a toxic metabolite of ethanol that causes the classical symptoms of headache, nausea, palpitation and facial flushing [2,4]. Elevation of acetaldehyde has also been shown to alter blood pressure in experimental animal models [5-7]. In addition to alcohol-metabolizing activity, recent research has identified ALDH2 as an enzyme responsible for the bioactivation of nitroglycerin in animals and human. ALDH2 catalyzes the conversion of nitroglycerin to 1,2-glyceryl dinitrate and nitrite, leading to cGMP production and smooth muscle relaxation [8,9]. These data suggests that ALDH2 is important for blood pressure regulation.

Consistent with these experimental studies, several cross-sectional or case–control association studies have reported a significant association between *ALDH2* SNPs and blood pressure or hypertension [10-19]. In this study, we aimed to examine whether common *ALDH2* genetic polymorphism predict development of hypertension in a prospective Chinese family cohort. We adapted a gene-based approach by systemically genotyping common tag SNPs capturing the *ALDH2* gene. We also explored potential gene-environmental interaction between *ALDH2* genotypes and various environmental risk factors.

## Methods

### The SAPPHIRe study cohort

The Stanford Asia-Pacific Program for Hypertension and Insulin Resistance (SAPPHIRe) was a collaborative study that was part of the Family Blood Pressure Program of the National Heart, Lung and Blood Institute of the National Institutes of Health and was designed to investigate the genetic determinants of hypertension and insulin resistance in the Chinese and Japanese individuals. The study collected sibling pairs who were either concordant or discordant for high blood pressure. Detailed descriptions of the study cohort were published in our previous work [20]. Hypertension was defined as systolic blood pressure ≧140 mmHg or diastolic blood pressure ≧90 mmHg or use of medications for high blood pressure. Individuals with pre-existing chronic illness such as diabetes, cancer, or diseases of the heart, liver, or kidney were excluded. The SAPPHIRe cohort study consisted of 6 field centers at baseline, including 4 field centers in Taiwan, one in Hawaii and one in Stanford University (San Francisco Bay area). A total of 1,143 subjects of Han Chinese descent from 392 families were enrolled at baseline. The follow-up study was conducted in the 4 Taiwan field centers and 753 individuals from 276 families were followed-up through the entire study. Other reasons of lost to follow-up included death (0.21%), lost of contact (5.7%), and rejection (16.56%). The Institutional Review Board of Tri-Service General Hospital in Taiwan, the National Taiwan University Hospital Research Ethics Committee, the Institutional Review Board of Taipei Veterans General Hospital, and the Institutional Review Board of Taichung Veterans General Hospital approved this study. Written informed consent was obtained from each participant.

### Clinical measurement

Blood pressure was measured using a mercury sphygmomanometer in 3 separate intervals at rest. Standardized interview-administered questionnaires were used to obtain information on demographic and lifestyle characteristics as previously described [21]. In brief, each participant was asked about smoking status and was categorized as non-smokers, ex-smokers or current smokers. Those who no longer smoked, but had smoked before were categorized as ex-smokers. The monthly alcohol consumption, grams of ethanol per day, was calculated based on the amount of usual ingestion of beer (5% ethanol), wine (12.5% ethanol), sake (16% ethanol) and liquor (40% ethanol) for each subject by a face-to-face interview. Light, modest, and heavy drinking was defined as average daily ethanol consumption 0–5 g, 5–10 g, and more than 10 g respectively [21]. Physical activity was assessed by recording the number of hours per day spent at each of five levels of activity [21].

### Selection of tag SNPs and genotyping

To identify common tag SNPs, we selected SNPs from the HapMap CHB (Chinese Beijing) database (HapMap Genome Browser release #24) (http://www.hapmap.org) using the Tagger program implemented in Haploview version 4 (http://www.broad.mit.edu/mpg/haploview/) with a minor allele frequency threshold of 0.1 and *r*^2^ of 0.8 [22,23]. Three tag SNPs that captured 100% of SNPs with minor allele frequencies more than 10% with mean maximal *r*^2^ = 0.962 were selected. The genotyping call rate was listed in Table 1. The concordance rate of this system based on 160 genotyping duplication was 99.38%.

**Table 1:** ***ALDH2*** **SNP information**

**Name**	**Minor/Major allele**	**Gene Region**	**HW** ***P***	**%Geno**	**MAF**
rs2238152	T/G	Intron	0.61	99.03	0.233
rs671	A/G	Exon Glu504Lys	0.23	99.21	0.261
rs2158029	G/A	Intron	0.68	99.38	0.343

HW P, Hardy –Weinberg P value; MAF, minor allele frequency; % Geno, genotyping call rate.

### Subjects for adipose tissue sampling

We additionally recruited 48 morbidly obese adults undergoing weight-loss surgery in Min-Sheng Hospital in Taiwan. Abdominal visceral adipose tissue was biopsied in a fasting state during surgery. Informed consent was obtained from each patient. The study was approved by the Institutional Review Board of Min-Sheng Hospital.

### Adipose tissue RNA extraction and reverse transcription (RT)

Adipose tissue was placed in liquid nitrogen immediately after resection and stored at −80°C until processed. Total RNA was extracted using REzol (Promega, Madison, WI) according to the manufacturer's instructions. Reverse transcription was performed using an RT kit (Promega, Madison, WI) with 1 μg of total RNA and 0.5 μg random hexamers in a final volume of 25 μl containing 200 U of Maloney murine leukemia virus reverse transcriptase, 20 nM dNTP, and 25 U of rRNasin for 1 h at 37°C. The reaction mixture was diluted to 100 μl with double-distilled water prior to PCR amplification.

### Quantification of mRNA expression levels by real-time PCR

A 5-μl sample of diluted cDNA was added to a mixture comprised of 10-μl 2x SYBR Master Mix Buffer to a final volume of 20 μL (Applied Biosystems, Foster City, CA). The pre-developed primers for *ALDH2* were PPH17047A-200 (QUIAGEN, Hilden, Germany). The primer sequence for *PPIA* (cyclophilin A) were:5' AGG TCC CAA AGA CAG CAG AAA AT 3' (forward) and 5' GTG AAA GCA GGA ACC CTT ATA ACC 3'(reverse). Thermocycling was done by 10 min at 95°C followed by 40 cycles of 30 sec at 95°C, 1 min at 60°C and 1 min at 72°C. Real-time quantitative PCR was analyzed by the ABI PRISM 7000 Sequence Detection System (Applied Biosystems, Foster City, CA). The fluorescent signal from each PCR reaction was collected as a peak-normalized value plotted versus the cycle number. Reactions were characterized by comparing the threshold cycle (Ct) values. Relative gene expression in relation to cyclophilin A RNA was calculated using the formula: ΔCt = (Ct of cyclophilin A) – (Ct of *ALDH2*).

### Statistical analysis

All data are expressed as mean values ± standard deviations (SD) unless otherwise specified. Tests for Hardy-Weinberg equilibrium were performed before marker-trait analysis. Pairwise LD measures D' and *r*^2^ were estimated to assess linkage disequilibrium (LD) between the SNPs in the *ALDH2* gene. The structure of the haplotype block was evaluated using the solid spine LD method implemented in the Haploview program [23]. Cox proportional hazard models were used to assess associations between the time to each progression event and the SNPs in the *ALDH2* gene. Hazard ratio (HR) for a genotype versus the reference genotype was estimated and 95% confidence intervals (CI) for these hazard ratios were also computed. We accounted for the correlations resulting from the sibship data in the modeling. To analyze the association of *ALDH2* genetic variants with quantitative traits, tests for population stratification and total association were performed using the quantitative transmission disequilibrium test (QTDT) program (http://www.sph.umich.edu/csg/abecasis/QTDT), which is based on a variance-components framework [24]. A test for total association was performed if there was no significant population stratification. The parameter estimates associated with each additional minor allele were estimated with generalized estimating equation. *P*-values were adjusted for age, sex, smoking and physical activity, and alcohol consumption. The study-wide significance threshold required for type 1 error less than 5% is estimated using the method proposed by Nyholt et al. which takes into account the LD between markers [25]. The population attributable fraction was calculated as follows: 1-{1÷[p^2^ HR_homo_ + 2p(1-p)HR_hetero_ + (1-p)^2^}, where p is the risk-allele frequency, HR_homo_ is the hazard ratio for homozygotes, and HR_hetero_ is the hazard ratio for heterozygotes. The association between the *ALDH2* genotype and *ALDH2* gene expression in adipose tissue was analyzed using non-parametric trend test implemented in STATA 9.0 (StataCorp LP, College Station, Texas).

## Results

### Characteristics of study subjects, allele frequencies, and LD structure

**Figure 1:**
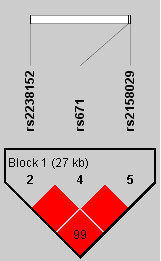
**LD between*ALDH2*SNPs in the SAPPHIRe cohort. Pairwise LD coefficients D' × 100 are shown in each cell (D' values of 1.0 are not shown).** The standard color scheme was applied for LD color display (LOD score ≥2 and D' =1 in bright red; LOD score ≥2 and D' <1 in blue; LOD score <2 and D' =1 in pink; LOD score <2 and D' <1 in white).

The basic clinical characteristics of participants at baseline are shown in Table 2. The nucleic acid composition, Hardy-Weinberg equilibrium test, genotyping call rate, and minor allele frequencies of selected SNPs are summarized in Table 1. The LD structure between SNPs is shown in Figure 1. All SNPs were within the same LD block. The allele frequencies of SNPs were similar to those in the HapMap CHB database [22].

**Table 2:** Characteristics of study participants

	**SAPPHIRe**	**Subjects for adipose tissue sampling**
N	1,134	48
Age (year)	48.39 ± 9.05	29.10 ± 8.24
Male sex (%)	46.83	14.89
BMI (kg/m^2^)	24.98 ± 3.44	40.93 ± 5.15
Waist circumference (cm)	83.59 ± 10.43	116.8 ± 10.79
Fasting glucose (mg/dl)	88.57 ± 10.61	105.3 ± 48.9
Fasting insulin	7.43 ± 5.58	21.6 ± 15.3
SBP (mmHg)	127.7 ± 24.15	124.6 ± 11.9
DBP (mmHg)	76.53 ± 13.38	76.3 ± 9.46
Triglycerides (mg/dl)	121.8 ± 76.25	214.8 ± 342.5
Total cholesterol (mg/dl)	185.9 ± 39.35	199.0 ± 31.1
HDL-cholesterol (mg/dl)	44.77 ± 12.23	43.61 ± 12.07
LDL-cholesterol (mg/dl)	117.59 ± 37.87	132.8 ± 33.97

Data are presented as mean ± S.D. BMI, body mass index; SBP, systolic blood pressure; DBP, diastolic blood pressure.

### SNP association with incidence of hypertension and changes of blood pressure during follow-up

Over an average follow-up period of 5.7 year, participants homozygous for the rs2238152 T allele (minor allele) had a higher risk of progression from non-hypertension to hypertension (HR = 2.88; 95% CI: 1.06-7.84; *P* = 0.03) than did non-carriers (Table 3). The genetic model was best fit with a recessive genetic mode and the corresponding population attributable risk fraction is ~7.1%. Participants homozygous for the mutant rs671 A allele had only a trend of increased hypertension incidence (HR = 2.13; 95% CI: 0.77-5.89; *P* = 0.15) compared to non-carriers. However, rs2238152 T allele was not significantly associated with changes of systolic/diastolic blood pressure (SBP/DBP) during follow-up. In contrast, the mutant rs671 A allele was significantly associated with increases of SBP (0.865 mmHg increase per year, *P* = 0.0071) and DBP (0.537 mmHg increase per year, *P* = 0.0026) during follow-up (Table 4).

**Table 3:** **Association of** ***ALDH2*** **genotypes with progression to hypertensions in SAPPHIRe cohort**

	**Incident cases/ 100 person-year (number of incident case)**			**Hazard ratio for Aa vs. AA (95%CI)**	***P***	**Hazard ratio for aa vs. AA (95%CI)**	***P***
	**AA**	**Aa**	**aa**				
rs2238152	3.84(27)	3.12(11)	5.47(4)	0.93 (0.46-1.87)	0.83	2.88 (1.06-7.84)	**0.03**
rs671	4.08(25)	3.02(13)	5.00(4)	1.08 (0.51-2.29)	0.84	2.13 (0.77-5.89)	0.15
rs2158029	4.11(21)	2.69(13)	5.72(8)	0.83 (0.4-1.7)	0.60	1.94 (0.79-4.77)	0.15

A: major allele; a: minor allele.

**Table 4:** Association of ALDH2 genotypes with changes of systolic/diastolic blood pressure in SAPPHIRe cohort

	**rs2238152**		**rs671**		**rs2158029**	
	**Estimate**	P*	**Estimate**	P*	**Estimate**	P*
Δ systolic blood pressure	0.0564	0.86	0.865	**0.0071**	0.269	0.38
Δ diastolic blood pressure	0.0316	0.87	0.537	**0.0026**	0.0241	0.89

Estimate: the changes in systolic/diastolic blood pressure associated with each additonal risk allele during follow-up.

* adjusted for age, sex, alcohol intake, smoking, drugs, and physical activity. Corrections of blood pressure for antihypertensive drugs (10/5 mmHg reduction per drug) was made.

### Interaction between environmental factors and SNPs on hypertension risk

**Figure 2:**
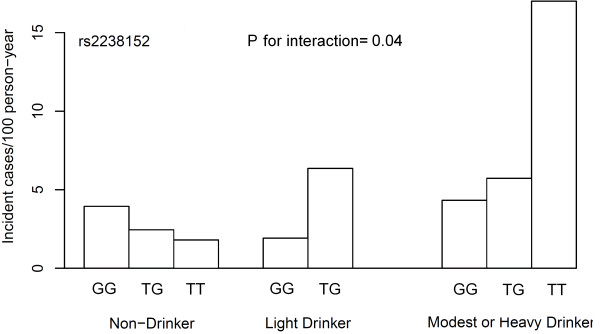
***ALDH2*** **genotype interaction with alcohol intake on progression to hypertension in the SAPPHIRe cohort.**

We next examined whether there was interaction between the *ALDH2* SNP and environmental factors on the risk of hypertension. The rs2238152 T allele was associated with higher incidence of hypertension in participants with moderate or heavy alcohol intake (incidence of hypertension: 4.32, 5.36, and 17.0 cases/person year for rs2238152 TT, TG, and GG genotype, respectively) (Figure 2). However, the genetic effect disappeared in participant who did not drink (incidence of hypertension: 3.94, 2.45, and 1.8 cases/person year for rs2238152 TT, TG, and GG genotype, respectively), indicating an interaction between SNPs and alcohol intake on the risk of hypertension (*P* for interaction = 0.04) (Figure 2). We did not detect significant interaction between *ALDH2* SNPs and other environmental factors including age, sex, smoking or physical activity on hypertension risk (data not shown).

### SNP association with ALDH2 mRNA in human adipose tissue

**Figure 3:**
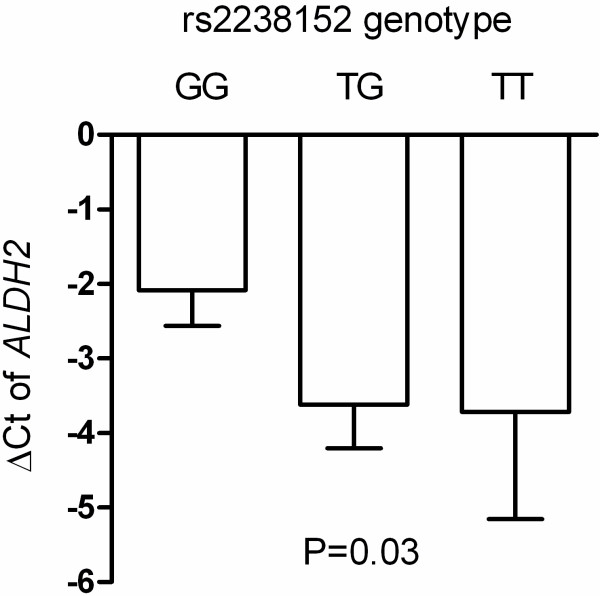
***ALDH2*gene expression level in abdominal adipose tissue according to rs2238152 genotype.** Relative gene expression in relation to cyclophilin A RNA was calculated using the formula: ΔCt = (Ct of cyclophilin A) – (Ct of *ALDH2*).

We further explored possible mechanism by which SNP rs2238152 affect clinical phenotypes. We measured *ALDH2* mRNA levels in human abdominal adipose tissue sampled from 48 adults. The rs2238152 T allele was associated with a significantly lower *ALDH2* gene expression level (*P* = 0.03) (Figure 3). Other SNPs were not associated with *ALDH2* gene expression level (Additional file 1: Figure S1).

## Discussion

In this study, we demonstrated that a common *ALDH2* genetic polymorphism was associated with progression to hypertension in a prospective Chinese cohort. The association was strongest in participants with heavy/moderate alcohol intake and was absent in participants who did not drink, indicating an interaction between *ALDH2* genetic variation and alcohol consumption on hypertension risk. To our knowledge, this is the first prospective cohort study demonstrating that *ALDH2* genetic polymorphism predicts development of hypertension in East Asian population.

Several cross-sectional studies and case–control association studies have consistently reported significant association between *ALDH2* genetic polymorphisms and blood pressure and hypertension in East Asian population [10-16]. A recent meta-analysis including 7,658 subjects determined that the rs671 A allele was associated with a significantly lower odds ratio of hypertension and lower blood pressure. In our study, rs671 was associated with a significant increase of blood pressure and a trend of increased hypertension risk during follow-up. Another adjacent SNP rs2238152, which was in complete LD with rs671 (D' = 1), was associated with increased risk for incident hypertension but was not associated with significant changes in blood pressure. The discrepancy between association with hypertension risk and changes of blood pressure may be partly attributed to the confounding of antihypertensive medication. In a recent large GWAS meta-analysis for hypertension in east Asian, Kato, et al. reported a strong association signal ~ 0.5 Mb downstream the *ALDH2* gene (*P* = 5.9 × 10^-13^)[18]. Daniel, et al. also reported strong association signal for diastolic blood pressure in the *SH2B3* gene (*P* = 1.6 × 10^-14^) in a GWAS for hypertension in Caucasian. The *SH2B3* gene is located in a large LD block spanning the *ALDH2* gene [19]. It is possible that the true causal variant is in LD with rs2238152 or rs671. Further fine mapping is needed to clarify the true causal variant.

We further explored the potential gene-environmental interaction between *ALDH2* variants and environmental factors on hypertension risk. Alcohol intake modified the genetic effect of *ALDH2* variant on hypertension risk. The mechanism underlying this interaction is currently unknown. Based on this observation, we propose that alcohol consumption level should be taken into account when *ALDH2* genetic information is used to predict further hypertension risk. We further demonstrated that the risk allele was associated with lower *ALDH2* gene expression in human adipose tissue. This suggested that the risk variant may influence the clinical phenotype though altered *ALDH2* gene expression.

Our study has 3 unique strengths. First, population stratification is still a common concern of case–control association studies, which may lead to false positive results. The family-based design of SAPPHIRe essentially eliminates all potential population stratification. Second, case–control studies are often confounded by recall bias or ascertainment bias. This study is a prospective cohort study with comprehensive records of baseline exposures and thus is free of such bias. Third, the environmental exposures were comprehensively recorded in the SAPPHIRe study, making a thorough exploration for gene-environment interaction possible.

This study also has some limitations. First, this study is relatively small with limited incident case so that a chance finding is not unlikely. The power of this study to detect variants with small effect is also limited. Given the hypertension prevalence of 25%[26] among the Chinese and a minor allele frequency of 0.25, the power to detect allelic odds ratios of 1.2, 1.5, and 2.0 for hypertension was 16.1%, 56.2%, and 95.1% respectively with type I error rate of 0.05. Second, the association between the rs2238152 genotype and progression to hypertension (*P* = 0.03) did not pass the significance threshold adjusted for multiple testing. For a type 1 error less than 5%, the study-wide significance threshold is estimated to be 0.02 after correction for the LD between each SNP [25]. However, previous cross-sectional or case–control studies had demonstrated significant association between *ALDH2* variants and hypertension. From a Bayesian point of view, the prior probability is already high and therefore stringent correction for multiple testing may not be necessary.

## Conclusion

In summary, we demonstrated that *ALDH2* genetic polymorphism predicted development of hypertension in a prospective cohort. Alcohol intake significantly modified the conferred risk. These data strengthen current evidence linking *ALDH2* genetic variants with hypertension.

## Competing interest

All authors declared that they have no competing interest.

## Authors’ contribution

YCC and YFC analyzed the data and drafted the manuscript. ITL, LTH, YJH, CAH, TQ, and TD designed the study and collected samples and clinical data. WJL and PCL collected surgical samples. CHC and DMR designed the study and helped to draft the manuscript. LMC designed the study, coordinated the experiments and drafted the manuscript. All authors read and approved the final manuscript.

## Source of funding

This work was supported in part by a grant from the Diabetes Fund of the National Taiwan University Hospital and the grants from the National Science Council of Republic of China (Taiwan) (NSC85-2331-B075-109Y, NSC86-2314-B075-099, NSC87-2312-B075-003Y; NSC 93-3112-B002-005), National Health Research Institutes in Taiwan (PH-100-PP03; PH-100-PP04), National Institute of Alcohol and Alcoholism (NIAAA11147), and the National Heart, Lung and Blood Institute (U01HL54527-0151).

## Pre-publication history

The pre-publication history for this paper can be accessed here:

http://www.biomedcentral.com/1471-2261/12/58/prepub

## Supplementary Material

Additional file 1 **Figure S1.***ALDH2* gene expression level in abdominal fat tissue according to rs671 (A) and rs2158029 (B) genotypes. Relative gene expression in relation to cyclophilin A RNA was calculated using the formula: ΔCt = (Ct of cyclophilin A) – (Ct of *ALDH2*). Click here for file
